# Secondary Sympatry Caused by Range Expansion Informs on the Dynamics of Microendemism in a Biodiversity Hotspot

**DOI:** 10.1371/journal.pone.0048047

**Published:** 2012-11-06

**Authors:** Romain Nattier, Philippe Grandcolas, Marianne Elias, Laure Desutter-Grandcolas, Hervé Jourdan, Arnaud Couloux, Tony Robillard

**Affiliations:** 1 Muséum national d’Histoire naturelle, Département Systématique et Evolution, UMR 7205 CNRS OSEB, Case postale 50 (Entomologie), Paris, France; 2 IMBE, Institut Méditerranéen de Biodiversité et d’Ecologie terrestre et marine, Aix-Marseille Université/CNRS/IRD/UAPV, UMR 237 IRD, Centre IRD de Nouméa, Nouméa, Nouvelle-Calédonie; 3 Genoscope, Centre national de Séquençage, Evry, France; University of California, Berkeley, United States of America

## Abstract

Islands are bounded areas where high endemism is explained either by allopatric speciation through the fragmentation of the limited amount of space available, or by sympatric speciation and accumulation of daughter species. Most empirical evidence point out the dominant action of allopatric speciation. We evaluate this general view by looking at a case study where sympatric speciation is suspected. We analyse the mode, tempo and geography of speciation in *Agnotecous*, a cricket genus endemic to New Caledonia showing a generalized pattern of sympatry between species making sympatric speciation plausible. We obtained five mitochondrial and five nuclear markers (6.8 kb) from 37 taxa corresponding to 17 of the 21 known extant species of *Agnotecous*, and including several localities per species, and we conducted phylogenetic and dating analyses. Our results suggest that the diversification of *Agnotecous* occurred mostly through allopatric speciation in the last 10 Myr. Highly microendemic species are the most recent ones (<2 Myr) and current sympatry is due to secondary range expansion after allopatric speciation. Species distribution should then be viewed as a highly dynamic process and extreme microendemism only as a temporary situation. We discuss these results considering the influence of climatic changes combined with intricate soil diversity and mountain topography. A complex interplay between these factors could have permitted repeated speciation events and range expansion.

## Introduction

Diversity hotspots in islands are characterized by very high specific richness and endemism resulting from local diversification of groups which are “trapped” in very limited spatial areas [1], [2], [3]. The theory behind this diversity rests upon the simple idea that local diversification occurred either by allopatric speciation, through the fragmentation of the limited space available into even more restricted daughter areas (microendemism), or by accumulation of daughter species without vicariance, through sympatric speciation based on non geographic isolation, for example involving ecological specializations [4], [5], [6].

Empirical case studies showed or suggested that diversifications in islands most often result from repeated events of local allopatric speciation and subsequent establishment of microendemism [7] due to Plio-Pleistocene climatic fluctuations resulting in rainforest fragmentation [8], [9], [10], or by the barriers made by large rivers [11], [12]. Contrasting with this general pattern, sympatric speciation has also been documented in islands [4], [5], [13], [14] and a few cases have been suspected in a context where distribution of species is restricted to small areas, i.e. microendemism [8], [15], [16], [17], [18], [19].

New Caledonia, one of most diverse island biodiversity hotspots [20], is no exception to the largely dominant allopatric pattern of distribution among related species, as shown in plants [21], [22], insects [8], [9], [23], land snails [24] and lizards [25], [26]. This suggests a major impact of allopatric speciation during the set up of microendemism in New Caledonia [27]. Yet, the relative importance of allopatric and sympatric speciation has not been addressed in many cases because of the low number of opportunities and the difficulty to distinguish between primary and secondary sympatry [7], [14], [28].

Here we analyse the mode, tempo and geography of speciation in the microendemic species of the cricket genus *Agnotecous* [29], [30]. This insect group shows a remarkable and generalised pattern of sympatry involving up to three sympatric species per location throughout New Caledonia (see Figure S1). Despite previous evidence for a major impact of allopatric speciation in the island, this pattern of distribution makes sympatric speciation plausible in this group.

We use a dated species-level phylogeny, analyses of diversification time-course, and age-range correlation methods to investigate the mode of speciation and causes of microendemism. We aim at discriminating between two alternative scenarios:

- Under a scenario of dominant allopatric speciation, the observed pattern of sympatry is caused by secondary sympatry with post-speciational movements.
- Under a scenario of dominant sympatric speciation, the observed pattern of sympatry results directly from recent sympatric speciation, with little or no post-speciational movements.

According to the dominant mode of speciation, we will compare and discuss the pattern of microendemism and tempo of species diversification in New Caledonia with respect to the influence of climatic changes combined with intricate soil distribution and mountain topography. This study will allow us understanding the origin of microendemic distributions in an evolutionary and biogeographical perspective.

## Materials and Methods

### Sampling and DNA Sequencing

The molecular sampling consists of 37 individuals corresponding to 17 out of the 21 known extant species of *Agnotecous*. Each species is represented by specimens known from 1–7 localities (mean = 1.8). Three species from different eneopterine tribes (*Nisitrus vittatus, Eneoptera guyanensis*, *Lebinthus santoensis*) and one species belonging to another cricket subfamily (*Acheta domesticus,* Gryllinae) were used as outgroups [30], [31]. For additional information on taxonomic sampling and sequencing protocols, see Tables S1 and S2, and Text S1 and S2.

### Phylogenetic and Dating Analyses

DNA sequences were aligned under Muscle with default parameters [32]. Individual datasets were constructed for the entire mitochondrial data, the entire nuclear data, and for the 28S and EF1α genes separately. Congruence tests between nuclear and mitochondrial datasets revealed a large extent of mitochondrial introgression (see Table S3, Figure S2 and Text S3), so only the nuclear data were used in the phylogenetic analyses. To obtain a species-level tree for *Agnotecous*, we generated a consensus sequence per *Agnotecous* species using IUPAC ambiguity codes under BIOEDIT v 7.0.9.0 [33]. Except slight differences in node supports, the phylogeny of the 16 consensus sequences does not reveal a different tree topology from the 37 specimens’ phylogeny (see Figure S3). For comparative purpose, we conducted Bayesian and Parsimony analyses on each dataset. Bayesian analyses used substitution models of evolution determined using the software MrModeltest v 2.3 [34], and selected using the Akaike Information Criteria [AIC; 35,36], and were performed in MrBayes 3.1.2 [37]. Parsimony analyses were performed under TNT [38]. For details about phylogenetic analyses, see Text S4.

Likelihood-ratio test [39] rejected rate homogeneity among taxa, advocating for a relaxed molecular clock model. To calibrate the trees, we used the results of a previous study [30] based on different combinations of five geological calibrations applied to a larger taxonomic scale. This study dated *Agnotecous* at 10.5 Myr ago, and its divergence with *L. santoensis* at 15 Myr ago, with standard deviations of 1 Myr for each calibration point [30]. We applied the Bayesian relaxed uncorrelated log normal approach implemented in BEAST v1.4.8 [40] to estimate the relative age of divergence of the lineages. We ran four Markov chains simultaneously for 15 million generations, sampling every 1000 generations to ensure the independence of samples. After checking for convergence a 10% burnin was applied, and the remaining posterior distribution of trees was summarized using TreeAnnotator [41], which retained the Maximum Clade Credibility (MCC) tree with branch length equal to the median lengths of all the trees (see Text S5 for additional BEAST parameters and details of calibrations).

### Analysis of Temporal Diversification

Lineage Through Time (LTT) plot derived from the BEAST MCC tree was used to visualise the time-course of diversification. We tested the null hypothesis of equal diversification rates across the *Agnotecous* phylogeny using the software SymmeTREE [ver. 1.1.; 42], which implements a whole-tree topological approach to the study of diversification rates. Diversification rate variation within the phylogeny is reported by a range of test statistics that vary in their sensitivity to nodal depth scales [42]. We then looked for the shift points by testing diversification rate change along branches using the function shift.test in the R package apTreeshape [43]. This test is based on the Δ1 statistic computed by SymmeTREE [44], and returns the probability of a diversification rate shift along the internal branch.

We also compare the fit of rate-variable models to the null hypothesis of constant diversification by using likelihood methods based on birth-death models and model-fitting approach (BDL) implemented in the R package LASER [45]. We tested for rate variation using (1) two rate-constant models: pure birth (pb) and birth death (bd), as null hypotheses and (2) one multiple-pure-birth rate model (y-2-rate), which detect 2 shift points, in otherwise constant speciation rates. Model selection was accomplished by comparing the difference in Akaike Information Criteria corrected for small sample sizes (AICrc) scores between the best rate constant and rate variable models as test statistic (ΔAICrc) [46] for the full data combined BEAST chronogram. For the bd and each non rate-constant model, the ΔAICrc statistic was compared with a distribution generated under the null hypothesis of rate constancy by the mean of 50000 trees generated under pure birth (yuleSim command in LASER), using the same number of extant and collected taxa as in the *Agnotecous* data set and using the estimated pure speciation value.

To compare diversification rates, we used APE [47], LASER [45] and GEIGER [48] packages for R environment. Diversification rates were also compared on all trees of the posterior distribution for different clades using the Yule model in APE. Four species are not included in the phylogeny but were taken into account with the rate.estimate function in GEIGER (details in Text S6). According to morphological studies [49], all these species belong to clade B: *A. petchekara* is close to *A. azurensis*, *A. humboldti* and *A. nekando* are close to *A. chopardi*, and *A. novaecaledoniae* is close to *A. sarramea* (within clade B2).

### Analysis of the Geographical Pattern of Speciation

Data on geographic distribution of the species were obtained from the collections of the following institutions: Muséum national d’Histoire naturelle (Paris), Natural History Museum (London), Queensland Museum (Brisbane), Osaka Museum of Natural History (Osaka), Naturhistorisches Museum (Vienna) and National Museum of Natural History (Leiden). The material examined in previous taxonomic studies of the genus was taken into account [29], [49], [50], [51], [52], [53], [54]. Species were recorded from a total of 52 localities for all the species (mean = 3.5; Table S4).

Distribution and range size of species were measured on a grid divided into quadrats of 0.025 degree intervals of latitude and longitude. For each spot from which a species was recorded, we considered an 8 km diameter circular area as the range size. This diameter was chosen based on the distribution of the most sampled species, *Agnotecous azurensis*, aimed at generating a continuous distribution area for this species. Considering these range sizes, our sampling covers 70% of *Agnotecous* main potential habitat (rainforest) (ca. 2670 km^2^ out of 3815 km^2^; after data available in DIVA-GIS v.7.1 [55] (see Figure S1).

To infer the predominant mode of speciation, and to account for post-speciational range changes, we applied the age-range correlation (ARC) method test [56]. A presence/absence matrix was used to calculate the degree of sympatry at each node of the tree. For pairs of terminal species, the degree of sympatry was calculated as the number of quadrats in which two species co-occur, divided by the number of quadrats occupied by the species with the smallest range [56], [57], [58], [59]. For deeper nodes in the tree we followed an alternative methodology using independent contrasts between pairs of taxa, which does not bring to artefactual increase of degree of sympatry through time [60], [61], [62], by acknowledging that unlike a phenotypic trait, distribution is an extrinsic feature of species that should not be interpreted only by mapping on a tree [63]. Average pairwise overlaps were calculated for each node. The degree of sympatry was plotted against the node ages estimated from BEAST analysis, and a linear regression was used to estimate intercept and slope. A positive slope with intercept <0.5 means that allopatric speciation predominates in the clade and that species become more sympatric as time since divergence increases, whereas a negative slope with intercept >0.5 means that sympatric speciation is the predominant mode and that overlap between species decreases over time [56], [57], [59], [60]. The statistical significance of the resulting slopes and intercepts was assessed via Monte Carlo simulation by random permutation of the overlap matrix to estimate a distribution under the null hypothesis of no phylogenetic signal. The fraction of randomized datasets with intercept and slopes greater than the observed data was calculated [60]. To conduct these analyses, we used the R package “phyloclim” v. 0.6 [64] with a modified function ARC provided by B. Fitzpatrick (pers. comm.).

Although *Agnotecous* is not characterised by high mobility (short-winged species unable to fly), we also computed two modified indicators following [65], known to be less sensitive to change in range size than the other indices: the proportion of cases where range overlap was 0 and the proportion of cases where range overlap was complete. These indicators were plotted against the node ages estimated from BEAST analysis, and a linear regression was used to estimate intercept and slope. A positive slope with intercept <0.5 means that allopatric speciation dominates, whereas a negative slope with intercept >0.5 means that sympatric speciation is the predominant mode [56], [60].

Finally, we computed the relationship between areas and ages of species to investigate post-speciational range expansion, and test whether microendemism is mostly found in younger clades.

Distribution areas and ages of clades were compared with the map of soil diversity using the GIS layers provided by Direction des Infrastructures, de la Topographie et des Transports Terrestres of the Gouvernement de la Nouvelle-Calédonie, and past climatic events with data in Chevillotte et al. [66].

## Results

### Phylogeny and Time-course of Diversification

**Figure 1:**
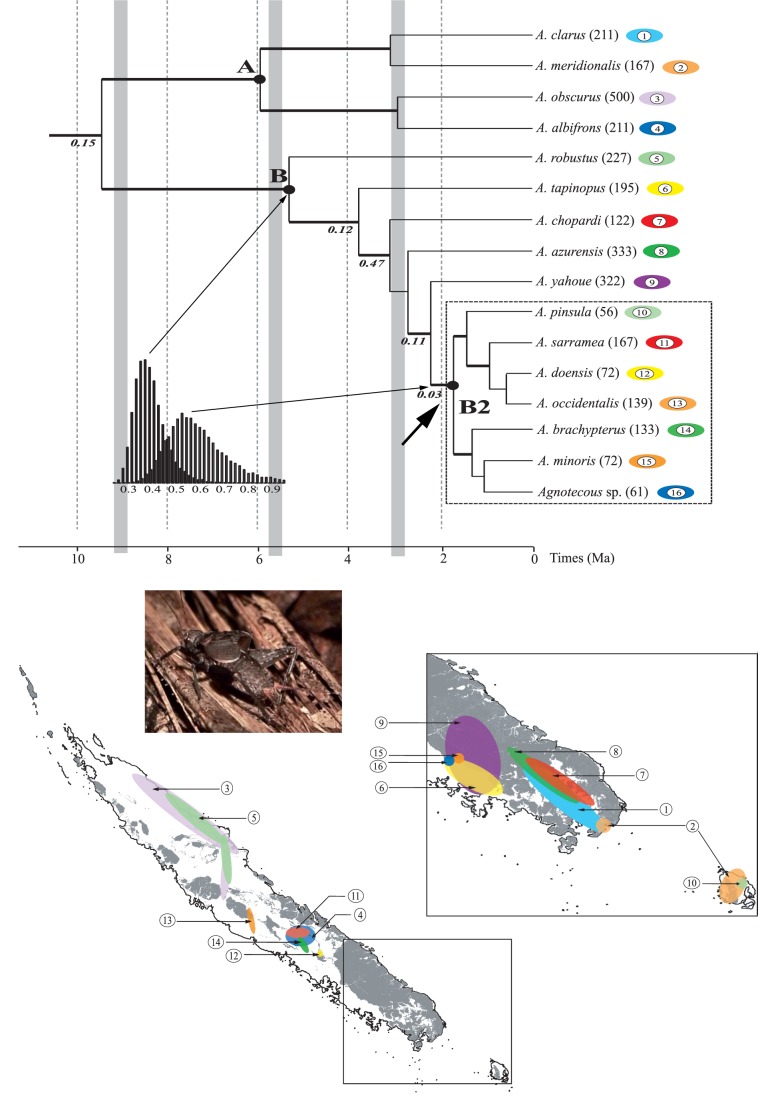
Chronogram reconstructed under BEAST and distribution of *Agnotecous* species. On the chronogram, branches shown as thick black lines indicate posterior probabilities ≥0.95. When different from 0, degrees of sympatry are indicated below branches. Grey vertical bars indicate morphogenesis episodes and dry periods (after [66]).The marginally significant case of diversification rate shifts detected with apTreeshape is indicate by an arrow, and the distribution of yule estimator of diversification for all trees the genus and clade B2 is shown. Hypothetical areas (in km^2^) are given on the right of the taxa names, and species are colored and numerated according their respective distribution. On the maps, the distribution of ultramafic rocks and corresponding metalliferous soils is indicated in grey (after [9]).

Two main lineages (A and B) of similar age (5.4 and 6 Ma, respectively) are recovered within the genus *Agnotecous* (Figure 1). Each clade consists of species distributed across New Caledonia. Major diversification events in *Agnotecous* are found around three major recent climatic events (Figure 1): The genus diversification started just before the first episode and was particularly high after the third episode, but the two main clades started diversifying during the second episode of dry and hot climate. In this context, clade B2 shows the highest diversification, which started just after the third episode of climatic changes and coincided with the peak of temperatures and the regular modifications of sea level.

**Figure 2:**
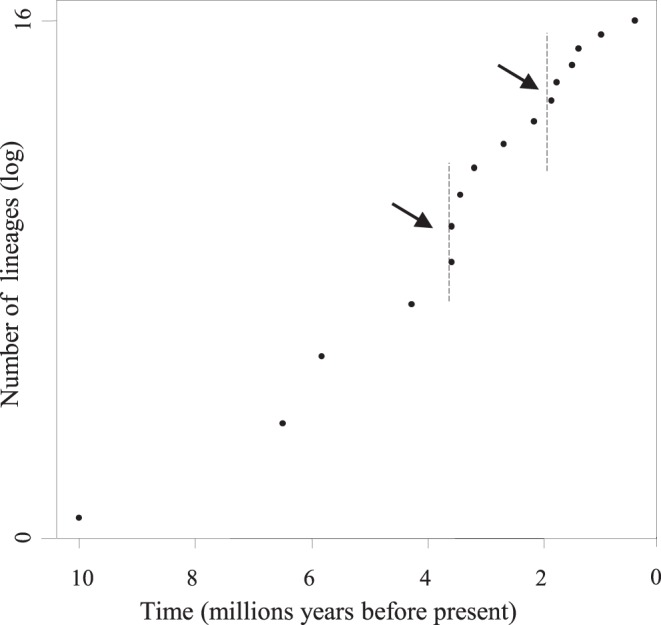
Species-level log lineages-through-time (LTT) plot for *Agnotecous* (excluding infra-specific data). Two minor increases in diversification rates around 3.5 Ma and 2 Ma are showing, but likelihood analyses reject the null hypothesis that diversification rates in *Agnotecous* have been constant through time.

The species-level LTT plots suggested two minor increases in diversification rates around 3.5 and 2 Myr ago (Figure 2), and a significant tree asymmetry was detected through the most to least sensitive test-statistics in SymmeTREE (0.0066<P<0.022), indicating that lineages diversified at significantly different rates. Although no significant diversification rate shifts were detected with apTreeshape, one marginally significant case was identified (p = 0.11) for the divergence of clade B2, i.e. ∼1.7 Myr ago. All details about these analyses are available in Tables S5–S6 and Figure S4.

These results are not confirmed by BDL analyses: the best rate-constant model (pure birth) had an AIC of 13.16 versus 15.97 for the rate-variable model (yule-2-rate) (ΔAICrc = 3.19) (see Table S7). Although marginally significant, the observed ΔAICrc statistics do not indicate a departure from the null hypothesis of rate constancy (p = 0.066) under a pure birth model. There is a marked difference of diversification rate (measured with ratio speciation: extinction of 0–0.5) of the clade B (0.33–0.29 without missing species and 0.39–0.34 including missing species) and clade B2 (0.72–0.62 without missing species and 0.80–0.69 including missing species). This difference is also observed on all trees of the posterior distribution (Figure 1).

### Geographical Pattern of Speciation

Geographical distributions reveal that sympatry is widespread amongst *Agnotecous.* Only three species belonging to clade B2 are not sympatric with any other (Figure 1). All highly microendemic species are located in the same clade (B2), which comprises 44% of the species and represents only 23% of the distribution area. Mann-Whitney tests show that these differences of area are highly significant: between clade B2 and other species (p = 0.004), and between clade B2 and all other species from clade B (p = 0.023). Species diversity and level of endemism are thus higher in the southern part of New Caledonia. Comparison of species distributions with soil diversity also shows that the southern region, where the highly endemic clade B2 occurs, is mostly characterized by metalliferous soils, while the rest of the species shows no relationship with the kind of soil.

**Figure 3:**
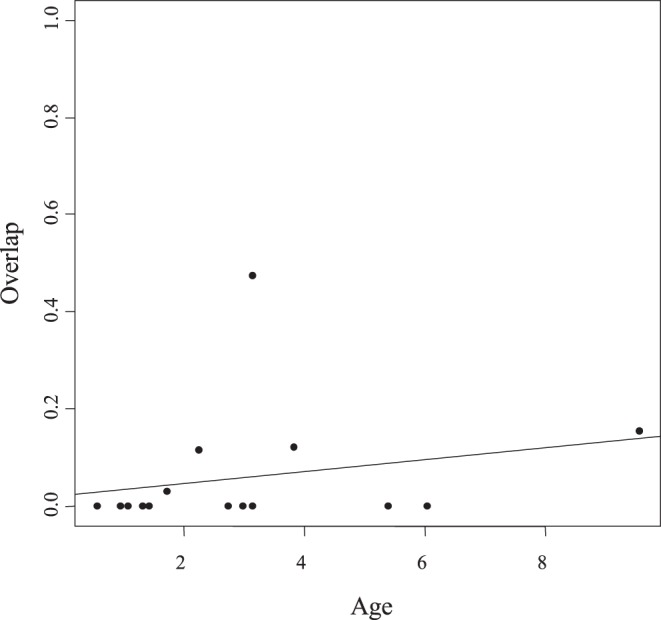
Degree of geographical overlap plotted as a function of relative age in the genus *Agnotecous*. Each point represents a node in the species phylogeny in Figure 1. Linear regression yielded low intercept (0.02170<0.5) and positive slope (0.01244), suggestive of allopatric speciation, but values are not statically significant (P-value = 0.462>>0.05).

**Figure 4:**
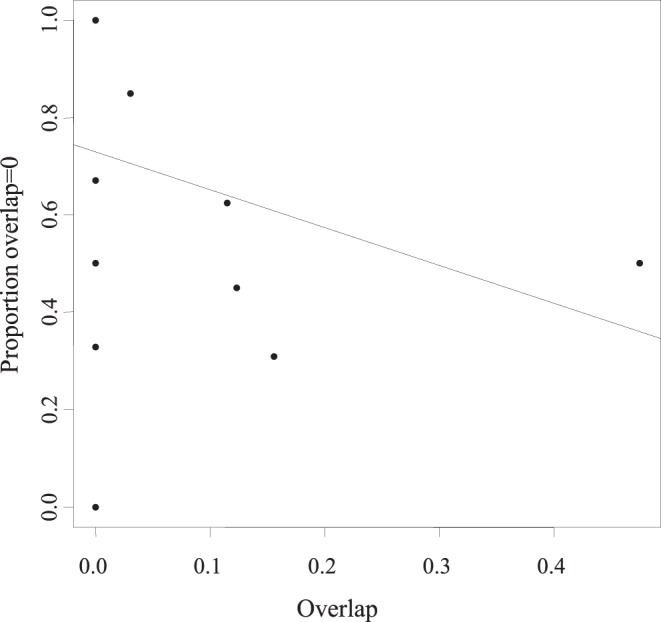
Correlation between the proportion of cases showing zero overlap and the proportion of range overlap. Using the Phillimore et al., 2008 method, allopatric speciation is suggestive but values are not statically significant (P-value = 0.2746>>0.05).

**Figure 5:**
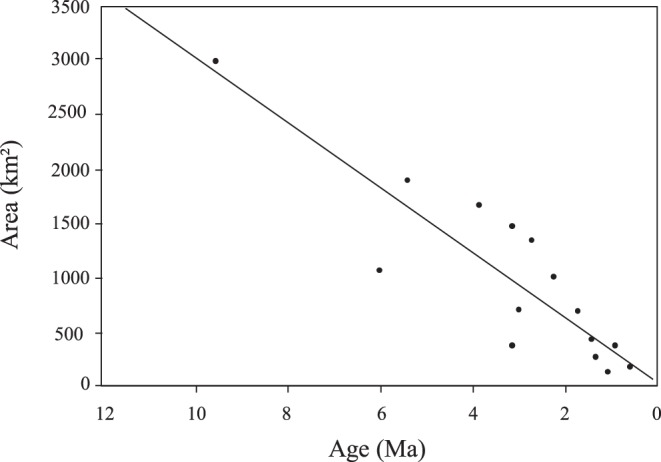
Relationship between areas and ages of species. The negative relationship between area and age of species indicates a geographical range expansion.

In the age-range correlation analysis, the regression of range overlap against node age in each clade generated positive intercept (0.02170<0.5) and slope (0.01244>0), thus rejecting a scenario of fully sympatric speciation (Figure 3). Values were not statistically significant (P = 0.462), meaning that a fully allopatric speciation model was not supported either. Similarly, the test on the proportion of cases where ranges overlap did not show a clear pattern (P = 0.2746), although here again the observed trend is closer to a model of allopatric speciation (Figure 4). More importantly, except for *A. minoris* and *Agnotecous sp.*, which are both microendemics, recently diverged sister species exhibit no geographical overlap, whereas more distantly related sister taxa do. This strongly suggests recent allopatric speciation. Finally, there is a negative relationship between area and age of species (Figure 5), indicating geographical range expansion.

## Discussion

In *Agnotecous*, a cricket genus characterized by restricted areas of distribution (microendemism; Figure 1) and complex patterns of sympatric species, the molecular phylogeny supports a hypothesis of dominant and recent allopatric speciation. ARC and range overlap tests clearly rule out a dominant sympatric speciation model (slope and intercept values typical of allopatric speciation), but they do not significantly support a fully allopatric speciation model. ARC tests are often non-conclusive [65], especially when assumptions of the test are not met, i.e. limited post-speciational range change and a single speciation mode. Still, this method highlights the relative importance between sympatric versus non-sympatric speciation and allows discussing them in relation with biological features and temporal diversification [62], [67]. In the *Agnotecous* case study (Figures 3 and 4), dominant allopatric speciation appears more likely than dominant sympatric speciation, at least in the last millions of years, when most of the diversification and microendemism took place. Closely related species are clearly allopatric and only two sister species show partially overlapping ranges (*A. minoris* and *Agnotecous* sp.). However, this case of partial sympatry is mostly a consequence of our definition of estimated range sizes, and these species are indeed not syntopic, but were found at different altitudes on the same mountain (Mont Mou: 375 m for *A. minoris* versus 1010–1140 m for *Agnotecous* sp.).

All the studies analysing the mode of speciation in New Caledonia have concluded that both the faunal and floral diversity originated from dominant allopatric speciation [27]. Despite the observed pattern of sympatry which first suggested sympatric speciation, our study is finally consistent with this general picture. What is then the origin of the microendemism and sympatric distributions observed in *Agnotecous?*

Recent events may have affected the post-speciational distributions of the species, resulting in secondary contacts between them. This kind of pattern was already found in other microendemic taxa showing lower levels of sympatry. For example, sympatric species occupy different microhabitats as a consequence of character displacement in Australian freshwater snails [68], or New-Caledonian Heteroptera [23].

Our results have highlighted that distribution patterns are related to the age of species: relatively old species (>3–4 Ma) show a larger distribution (>200 km^2^), and younger species show more restricted distributions. This is especially true in the clade B2 (Figure 1), which contains 44% of the species in only 23% of the total area of distribution in the southeast of New Caledonia (Figure 1). Clade B2 is dated at 2.8 Myr ago only and includes 7 species showing no mutual sympatry, with putative range size comprised between 56–167 km^2^ (mean = 100 km^2^), compared to the 122–500 km^2^ for other species (mean = 253 km^2^). Highly microendemic species are thus clearly the most recent ones (Figure 1). In other words, correlation between size of distribution area and species age could be considered as a strong indication of range expansion after speciation events. In this view, extreme microendemism may be seen as a transitory step in the history of a species distribution, that is only observed in the case of the youngest species. The whole pattern, allopatric speciation and evolution toward secondary sympatry, is geographically very coherent, even though the most recent events of speciation date back to more than 1 Myr. It emphasizes once more that the geographical study of speciation depends on the stability of distributional patterns associated with speciation, that should not be obscured by too large and frequent events of range change or extinctions [69].

If generalised through relevant testing in other groups of organisms in other regions, this hypothesis may explain the extraordinary level of microendemism in some megadiverse hotspots. The question may then become not only why are there so many species with such small distribution areas, but why speciation and distributional patterns remain so stable without immediate range expansion. As a matter of consequence, range size of endemic species cannot only be assumed to be controlled by a given set of classical influential factors, but should also be considered a possible by-product of evolutionary time.

Several factors have been considered to explain high speciation rates and microendemism in biodiversity hotpots such as New Caledonia [27], Madagascar [28], Mexico [70] or Eastern North America [71]. Predominantly, altitude and climatic variations have been hypothesized to promote microendemic diversification as a consequence of climatic variations, through allopatric speciation with niche conservatism on different mountains [72]. Coupled with recent climatic changes, landscape complexity may be another factor involved in speciation. For example, in Malagasy endemic cophyline frogs, mountains constitute key areas for endemic diversity because of the presence of slopes, which increase community complexity and favour parapatric or sympatric speciation, while the altitudinal ranges promote allopatric speciation through topogeographic opportunities [28]. In New Caledonian cockroaches *Lauraesilpha*, speciation of microendemic species is explained by isolation on different mountains following climatic variations [9], [73].

In our study, there is no clear correspondence between climatic events [66] and species diversification (Figure 1). The initial diversification in *Agnotecous* took place during a climatic episode going from 16 to 4 Myr ago. This time lag, similar to that found in Trichoptera from New Caledonia [17], involves a period of long-term climate change and modification of the landscape with four successive planation episodes and dry periods, accompanied by oscillations of continental temperature and sea level [66]. In more recent taxa (after 3 Myr ago), we observe a marked difference of diversification rates (between clades B and B2) co-occurring with a period of climate change marked by frequent changes of sea level between −50 m and 60 m [66]. It suggests that the influence of climatic changes on speciation processes could be mediated by another factor.

Intense fragmentation of soil and habitats caused by climatic events may explain the pattern of distribution observed in *Agnotecous*. Soil diversity and especially metalliferous soils have often been seen as primarily important for speciation in New Caledonia [74], but the role of metalliferous soils has only been documented in plants [e.g. 75] and in one group of aquatic insects [76]. Metalliferous soils derived from ultramafic rocks almost covered the entire surface of New Caledonia after the Eocene obduction, and they still cover about one third of the territory today, mostly as a dense patchwork in the southeast, with addition of patches in the north-west [66], [77]. Ultramafic rocks are rich in ferromagnesian minerals (e.g. olivines, amphiboles and pyroxenes), which are easily altered by the action of water and wind [78]. Such alteration is responsible for the release of large quantities of iron, magnesium and nickel, and results in the complex landscape which is found in ultramafic massifs of New Caledonia [66]. This chemical process means that recent climatic changes may have affected these regions with metalliferous soils more intensely than nearby regions without these soils, especially during periods of alteration/erosion/peneplanation. This would result in range fragmentation with more complex landscape and habitat reduction, which in turn would favour recent allopatric speciation.

*Agnotecous* species are found both on metalliferous and non metalliferous soils, without a clear and contrasted radiation on one kind of soil (same conclusions as in [9]). Nevertheless, most species of the younger clade B2, i.e. species with restricted distribution areas, occur in the south, on or nearby metalliferous soils and ultramafic rocks (Figure 1). Species of clade A and of the first lineages of clade B mostly occur far from ultramafic rocks, and they have larger distribution ranges, especially in northern New Caledonia (Figure 2). As allopatry is the dominant mode of speciation in *Agnotecous*, soil heterogeneity may have an indirect effect on habitat fragmentation, which could in turn explain local microendemism concentrated in the south of the island.

### Conclusion

Even in the *Agnotecous* lineage showing a generalized pattern of sympatry, speciation appeared predominantly allopatric. Therefore, our study brings new and conservative evidence in favour of the classical scenario of allopatric speciation leading to diversification in biodiversity hotspots. Our study also suggests that extreme local diversity may be explained by the recent establishment of microendemism through an environment-driven process. In this context, speciation studies help understanding that present-day microendemism is a first stage of a complex and dynamic process. Future studies should be conducted to specify the possible complex interplay between climatic changes, mountain complexity and soil-driven landscape changes, which could have permitted repeated speciation events and range changes.

## Supporting Information

Figure S1 Geographic distribution of *Agnotecous* species showing the general pattern of sympatry throughout New Caledonia.
(PDF)

Figure S2 Topologies obtained in parsimony (a) and Bayesian inference (b) for mitochondrial dataset only.
(PDF)

Figure S3 Comparison between the phylogeny obtained from the 37 specimens (a, c) and the phylogeny from the 16 consensus sequences (b, d).
(PDF)

Figure S4 Repartition of p-values obtained by the analyses under apTreeshape.
(PDF)

Table S1 Additional information on taxonomic sampling, voucher references (specimen numbers in MNHN collections) and GenBank accession numbers of the specimens included in this study.
(PDF)

Table S2 Primers used in this study.
(PDF)

Table S3 Results of the Bayesian test of monophyly for *Agnotecous* species.
(PDF)

Table S4 Details of the geographic distribution of species used in the analysis of geographical pattern of speciation.
(PDF)

Table S5 Results of the asymmetry tests conducted under SymmeTREE for seven test statistics.
(PDF)

Table S6 Results of the diversification rate change test conducted under the R package apTreeshape.
(PDF)

Table S7 Results of the BDL analyses and comparison of AIC values from different diversification models.
(PDF)

Text S1 Additional information on sequencing protocols.
(PDF)

Text S2 Characteristics of mitochondrial (a) and nuclear (b) datasets used in this study.
(PDF)

Text S3 Congruence tests between nuclear and mitochondrial data sets.
(PDF)

Text S4 Details about phylogenetic analyses.
(PDF)

Text S5 Additional BEAST parameters and details of calibrations.
(PDF)

Text S6 Details of the rate.estimate function used in GEIGER.
(PDF)
