# Supplementary figures and images for: Secondary Sympatry Caused by Range Expansion Informs on the Dynamics of Microendemism in a Biodiversity Hotspot
Source: PLoS One. 2012 Nov 6;7(11):e48047. doi: 10.1371/journal.pone.0048047 (PMC3490955; doi:10.1371/journal.pone.0048047)

Figure S1

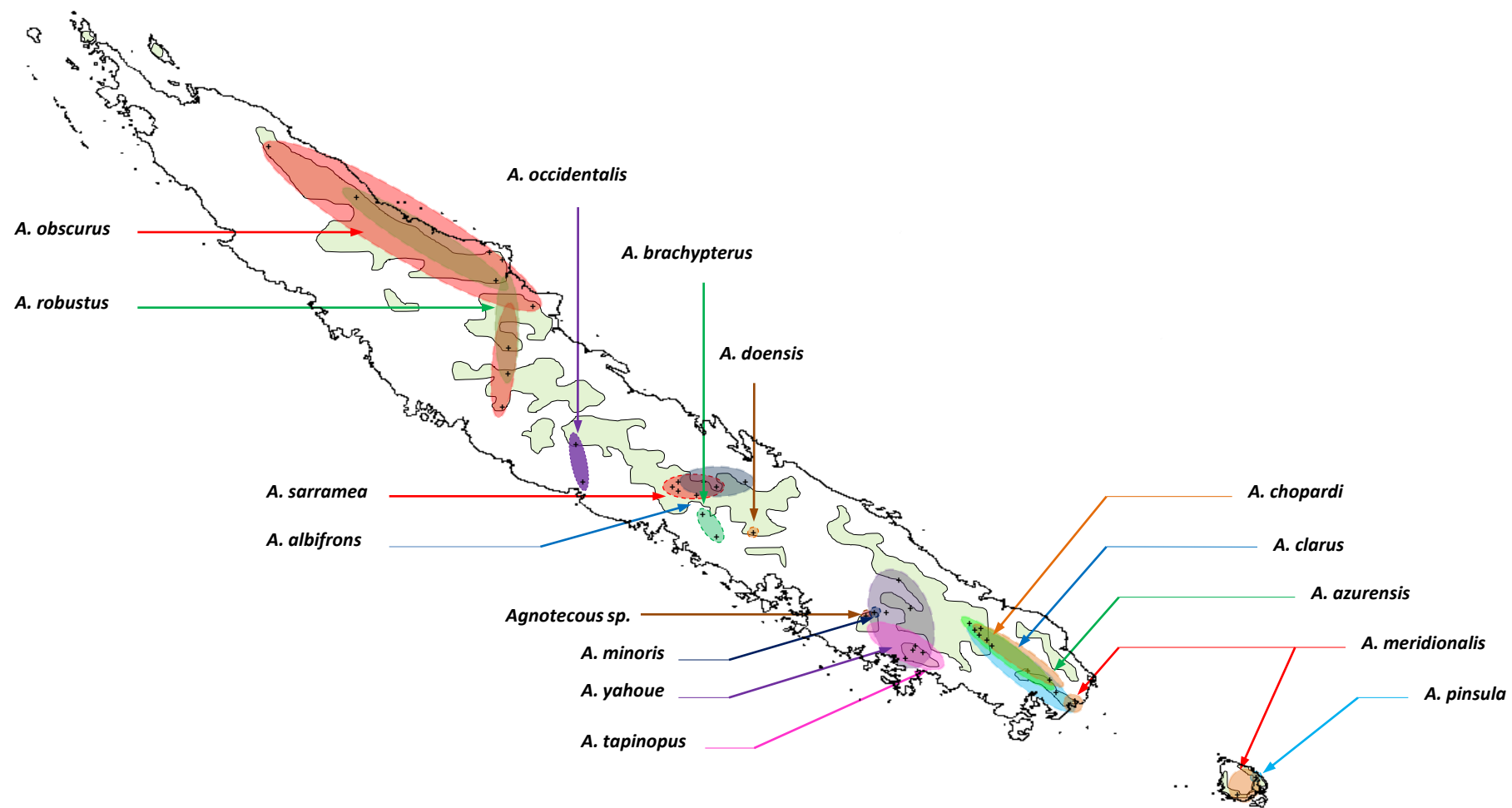

Supplement: Figure S1 — Geographic distribution of Agnotecous species showing the general pattern of sympatry throughout New Caledonia. (PDF) [file pone.0048047.s001.pdf]

(a)

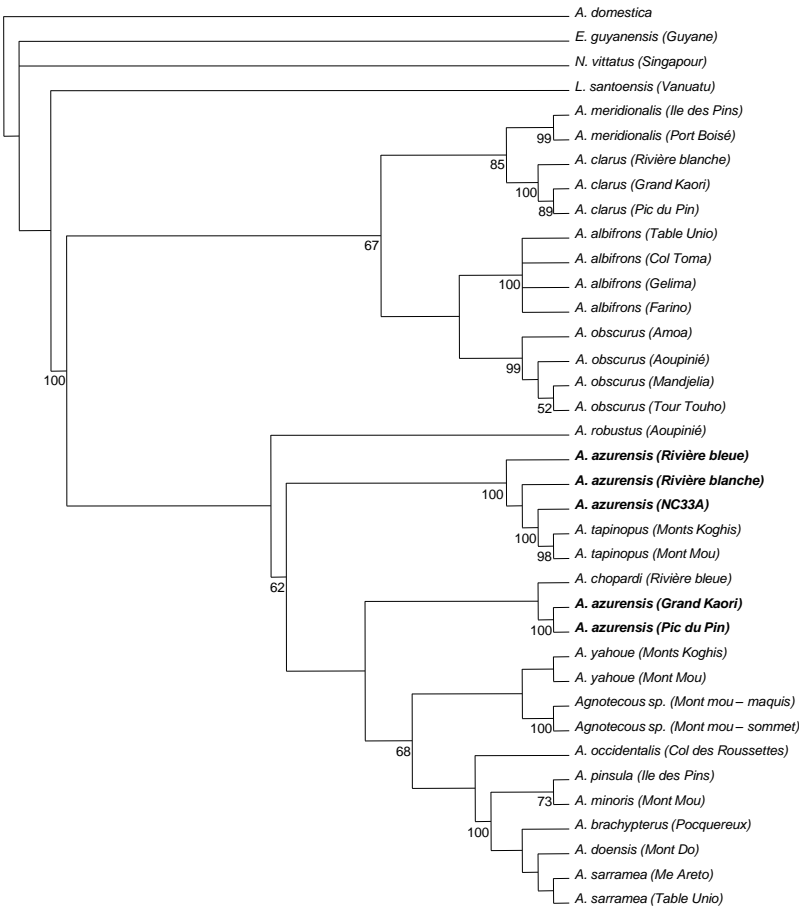

(b)

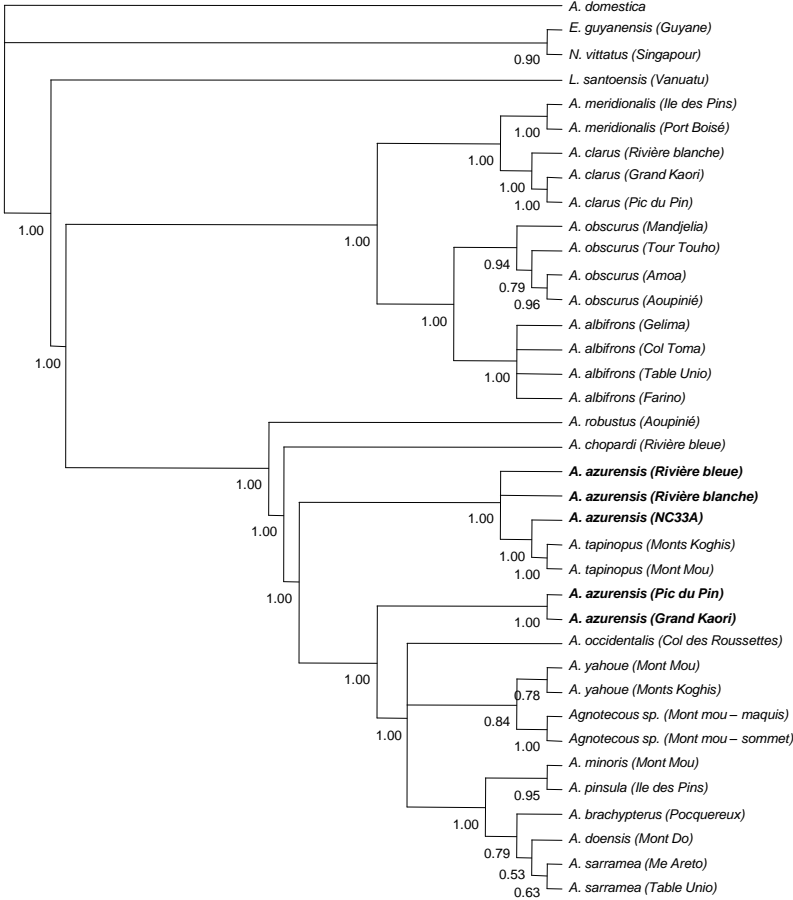

Figure S2

Supplement: Figure S2 — Topologies obtained in parsimony (a) and Bayesian inference (b) for mitochondrial dataset only. (PDF) [file pone.0048047.s002.pdf]

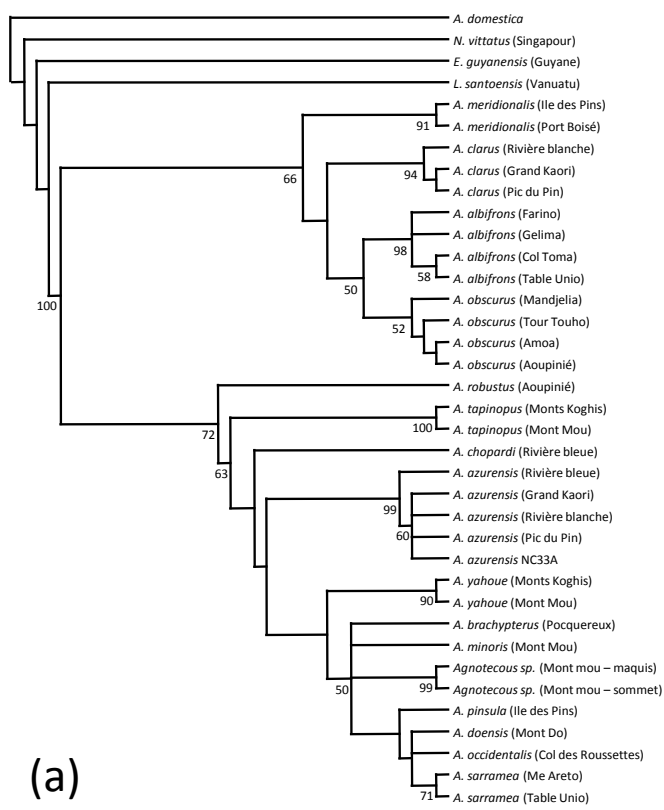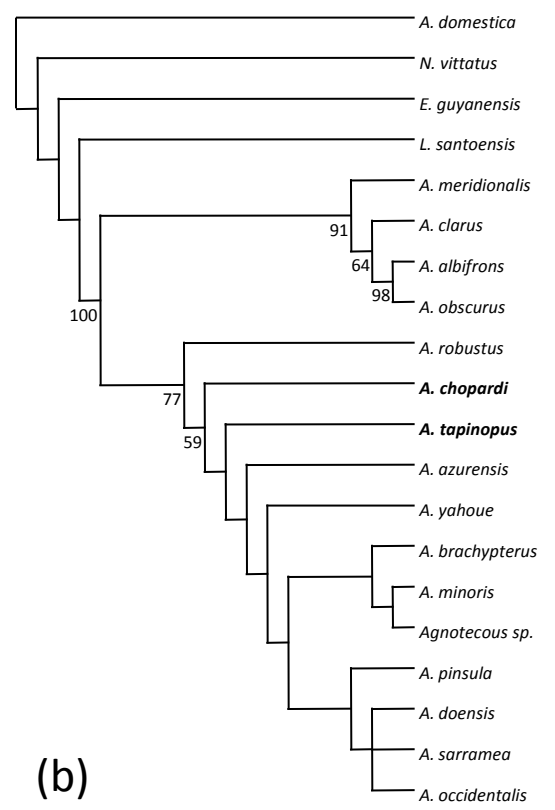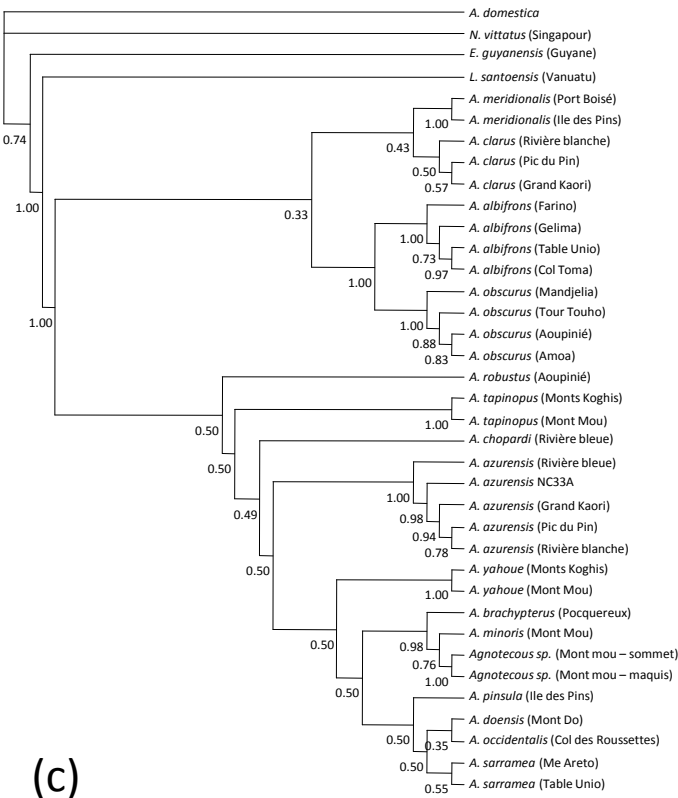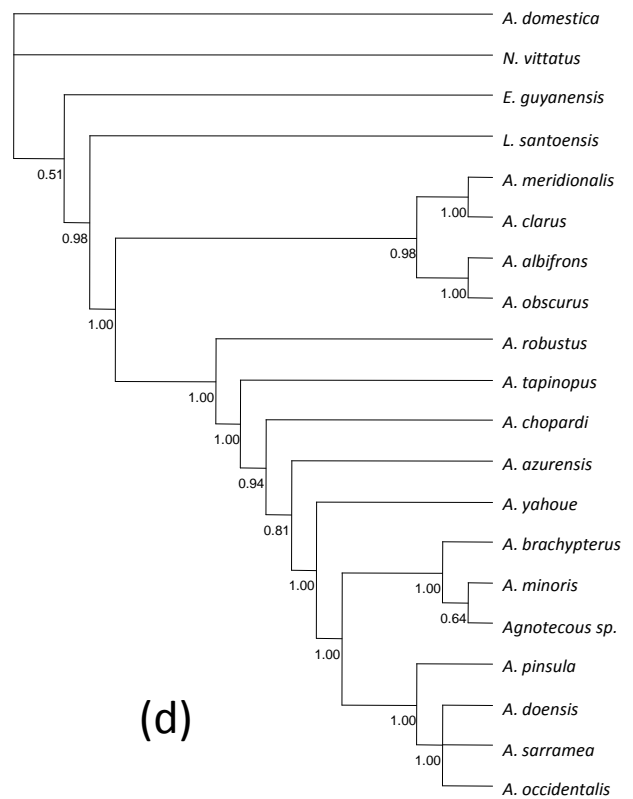

Figure S3

Supplement: Figure S3 — Comparison between the phylogeny obtained from the 37 specimens (a, c) and the phylogeny from the 16 consensus sequences (b, d). (PDF) [file pone.0048047.s003.pdf]

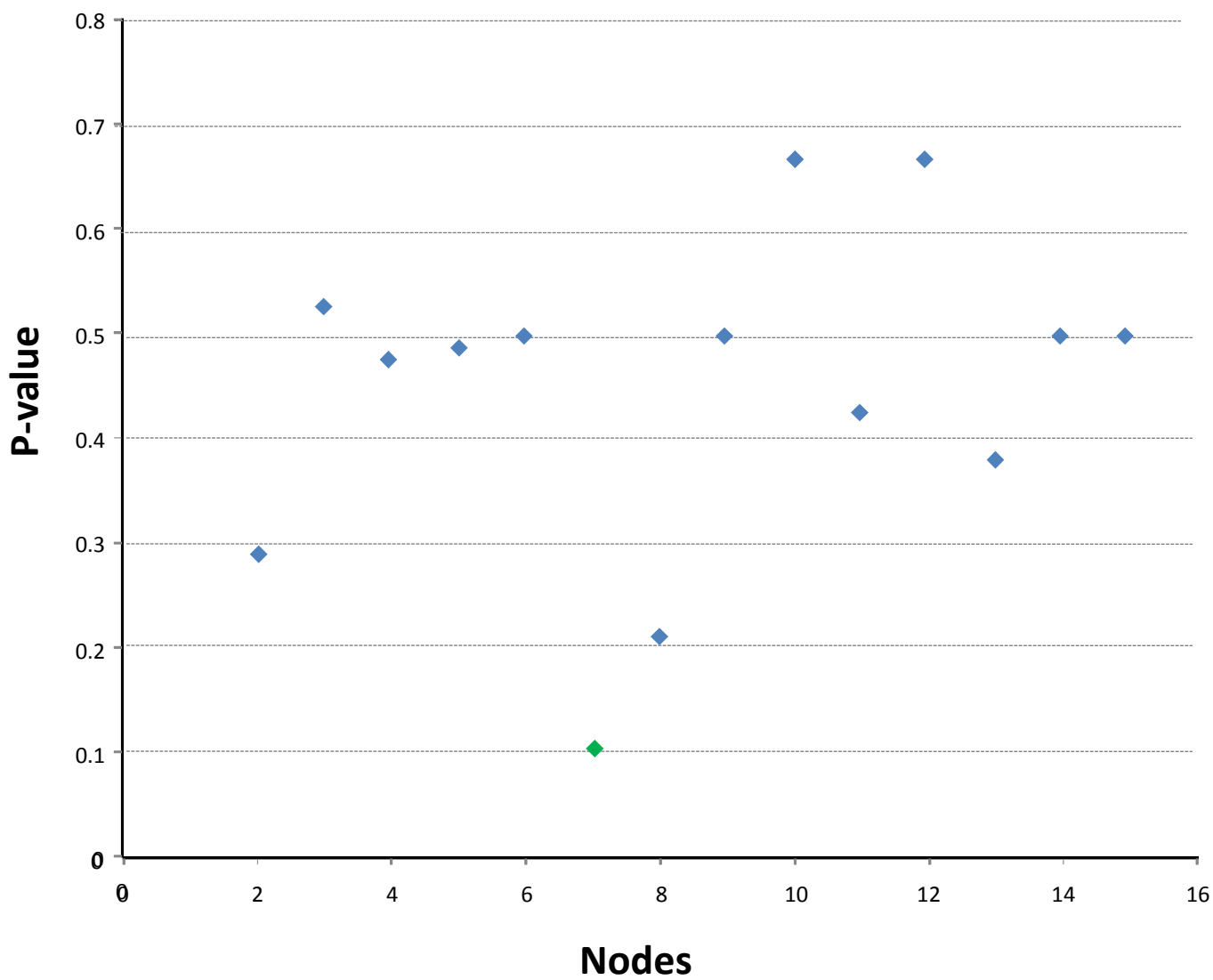

**Figure S4**

Supplement: Figure S4 — Repartition of p-values obtained by the analyses under apTreeshape. (PDF) [file pone.0048047.s004.pdf]
