# Supplementary material for: Secondary Sympatry Caused by Range Expansion Informs on the Dynamics of Microendemism in a Biodiversity Hotspot
Source: PLoS One. 2012 Nov 6;7(11):e48047. doi: 10.1371/journal.pone.0048047 (PMC3490955; doi:10.1371/journal.pone.0048047)
Supplement: Table S1 — Additional information on taxonomic sampling, voucher references (specimen numbers in MNHN collections) and GenBank accession numbers of the specimens included in this study. (PDF) [file pone.0048047.s005.pdf]

TABLE S1

Species	Locality	Voucher specimens	GenBank accession number									
			MITOCHONDRIAL					NUCLEAR				
		MNHN collection number	16S	12S	Cytb	CO1	CO2	28S	EF1a	Hexk	H3	18S
Agnotecous yahoue **Otte, 1987**	*Mont Mou (basis)*	*MNHN-ENSIF-2773*	JX897367	JX897388	JX897337	JX897437	JX897462	JX897479	JX897501	-	-	-
	Monts Koghis	*MNHN-ENSIF-2766*	JX897366	JX897387	JX897334	JX897438	JX897463	JX897478	JX897500	JX897542	JX897549	JX897602
Agnotecous sp.	*Mont Mou (summit)*	*MNHN-ENSIF-2774*	JX897364	JX897385	JX897336	JX897435	JX897464	JX897482	JX897518	-	JX897563	JX897603
	Mont Mou (maquis)	*MNHN-ENSIF-2751*	JX897365	-	JX897312	JX897436	-	JX897481	JX897517	-	-	-
Agnotecous azurensis **Desutter-Grandcolas, 2006**	*Pic du Pin*	*MNHN-ENSIF-2778*	JX897361	JX897390	JX897332	JX897427	JX897455	JX897473	JX897506	-	JX897568	JX897594
	Rivière Bleue (Pourina)	*MNHN-ENSIF-2780*	JX897358	JX897376	JX897329	JX897423	JX897453	JX897475	JX897502	-	JX897566	JX897595
	Rivière bleue	*MNHN-ENSIF-2789*	JX897374	JX897377	JX897330	JX897425	-	JX897476	JX897504	JX897539	JX897567	JX897600
	Rivière Blanche	*MNHN-ENSIF-2779*	JX897346	-	JX897328	JX897424	-	JX897474	JX897503	JX897532	JX897569	-
	Grand Kaori	*MNHN-ENSIF-2777*	JX897360	JX897389	JX897331	JX897426	-	JX897472	JX897505	-	JX897565	JX897593
Agnotecous brachypterus pocquensis **Desutter-Grandcolas, 2010**	*Pocquereux*	*MNHN-ENSIF-2664*	JX897370	JX897382	JX897341	JX897432	JX897459	JX897485	JX897516	JX897530	JX897571	JX897596
Agnotecous sarramea **Desutter-Grandcolas, 1997**	*Mé Aréto*	*MNHN-ENSIF-2764*	JX897372	JX897380	JX897342	JX897430	JX897456	JX897471	JX897511	JX897538	JX897561	JX897598
	Table Unio	*MNHN-ENSIF-2787*	JX897371	JX897384	JX897339	JX897433	JX897458	JX897477	JX897514	-	JX897560	JX897604
Agnotecous pinsula **Robillard, 2010**	*Ile des Pins*	*MNHN-ENSIF-2624*	JX897369	JX897383	JX897338	JX897429	JX897457	JX897484	JX897509	-	JX897562	JX897599
Agnotecous chopardi **Desutter-Grandcolas, 2006**	*Haute Rivière bleue*	*MNHN-ENSIF-2781*	JX897363	JX897391	JX897335	JX897411	JX897440	JX897469	JX897499	JX897537	JX897559	JX897601
Agnotecous doensis **Desutter-Grandcolas, 2006**	*Mont Do*	*MNHN-ENSIF-2782*	JX897368	JX897381	JX897340	JX897431	JX897460	JX897480	JX897513	JX897536	JX897558	JX897592
Agnotecous tapinopus **Saussure, 1878**	*Mont Mou*	*MNHN-ENSIF-2769*	JX897345	JX897379	JX897326	JX897421	JX897452	JX897468	JX897508	JX897541	JX897557	JX897580
	Monts Koghis	*MNHN-ENSIF-2770*	JX897344	JX897378	JX897327	JX897422	JX897454	JX897470	JX897507	JX897540	JX897556	JX897605
Agnotecous albifrons **Desutter-Grandcolas, 1997**	*Gelima*	*MNHN-ENSIF-1771*	JX897354	JX897396	JX897316	JX897416	JX897445	-	JX897529	-	JX897574	JX897585
	Farino	*MNHN-ENSIF-1766*	JX897351	JX897395	JX897315	JX897417	JX897444	-	JX897526	-	JX897575	JX897584
	Table Unio	*MNHN-ENSIF-1770*	JX897352	JX897397	JX897317	JX897419	JX897447	-	JX897528	-	JX897573	JX897581
	Col Toma	*MNHN-ENSIF-2767*	JX897353	JX897394	JX897314	JX897418	JX897446	JX897490	JX897527	-	JX897572	JX897583
Agnotecous obscurus **(Chopard, 1970)**	*Aoupinié*	*MNHN-ENSIF-2786*	JX897356	JX897398	JX897319	JX897415	JX897449	-	JX897510	-	-	JX897591
	Mandjelia	*MNHN-ENSIF-2785*	JX897357	JX897393	JX897320	JX897412	JX897450	JX897487	JX897525	JX897531	JX897576	JX897587
	Touho	*MNHN-ENSIF-2784*	JX897355	JX897399	JX897321	JX897413	-	-	JX897524	-	JX897578	JX897582
	Amoa	*MNHN-ENSIF-2783*	-	JX897392	JX897318	JX897414	JX897448	-	JX897497	-	JX897577	JX897586
Agnotecous meridionalis **Desutter-Grandcolas, 2006**	*Port Boisé*	*MNHN-ENSIF-2771*	JX897350	JX897402	JX897313	JX897410	JX897442	JX897489	JX897520	-	JX897550	JX897597
	Ile des Pins	*MNHN-ENSIF-2772*	JX897349	JX897401	JX897311	JX897420	-	JX897488	JX897519	JX897545	JX897553	JX897579

***Agnotecous robustus* (Chopard, 1915)**	*Aoupinié*	*MNHN-ENSIF-2752*	JX897359	JX897375	JX897333	JX897406	JX897443	-	JX897498	JX897535	JX897555	JX897588
***Agnotecous clarus* Desutter-Grandcolas, 2006**	*Rivière Blanche*	*MNHN-ENSIF-2763*	JX897348	-	JX897325	JX897408	-	JX897486	JX897521	-	JX897552	-
	Grand Kaori	*MNHN-ENSIF-2776*	JX897343	-	JX897323	JX897409	-	JX897491	JX897522	JX897543	JX897551	-
	Pic du Pin	*MNHN-ENSIF-2788*	JX897347	JX897400	JX897324	JX897407	JX897451	JX897492	JX897523	JX897544	JX897554	JX897590
***Agnotecous occidentalis* Desutter-Grandcolas, 2006**	*Col des Roussettes*	*MNHN-ENSIF-2765*	JX897362	JX897386	JX897322	JX897434	JX897461	-	JX897512	JX897533	JX897570	JX897589
***Agnotecous minoris* Robillard, 2010**	Mont Mou (basis)	*MNHN-ENSIF-1389*	JX897373	-	-	JX897428	-	JX897483	JX897515	JX897534	JX897564	-
***Lebinthus santoensis* Robillard, 2009**	Vanuatu, Espiritu Santo	*MNHN-ENSIF-2437*	JF972527	JF972511	JF972495	JX897405	JX897441	JX897467	JX897496	-	JX897548	JF972542
***Nisitrus vittatus* (Haan, 1842)**	Singapore	*MNHN-ENSIF-2742*	AY905314	AY905284	AY905369	-	-	-	JX897493	-	JX897546	AY905340
***Eneoptera guyanensis* Chopard, 1920**	French Guiana	*MNHN-ENSIF-2741*	AY905301	AY905272	AY905355	JX897404	-	JX897466	JX897495	-	JX897547	AY905331
***Acheta domesticus* (Linnaeus, 1758)**	Worldwide distribution	-	AF248698	ADZ97611	AF248682	JX897403	JX897439	JX897465	JX897494	-	-	AD18SITS1
