# Supplementary material for: Secondary Sympatry Caused by Range Expansion Informs on the Dynamics of Microendemism in a Biodiversity Hotspot
Source: PLoS One. 2012 Nov 6;7(11):e48047. doi: 10.1371/journal.pone.0048047 (PMC3490955; doi:10.1371/journal.pone.0048047)
Supplement: Table S2 — Primers used in this study. (PDF) [file pone.0048047.s006.pdf]

TABLE S2

	Gene	Name	Sequencing primers (5' - 3')	Reference	Annealing temperature
MITOCHONDRIAL	**CO1**	L2	GCAACGATGATTATTTTCCACT	modified from [15]	48-50°C
		H2	CCTGGTAAAATTAGAATGTAAACTTCTG		
		A+GR	CCAGTTTTAGCTGGTGCTATTACIAT	[16]	47°C
		B-GR	TAATCRGAATATSGTCGTGGTATTCC		
	**CO2**	co2a	GGTCAAACAATTGAGTCTATTTGAAC	[17]	55°C
		co2e	CCACAAATTTCTGAACATTGACCA		
		RNa	TACWMAYCGWTTYCTTYTTGAAGGWC	modified from [17]	47°C
		RNe	CGACCTGGAGTTGCATCAG		
	**Cytb**	427F	YTWGTWCAATGARTMTGAGG	[18]	48°C
		800R	CCYARTTTATTAGGAATTGATCG		
	**12S**	F	TACTATGTTACGACTTAT	[19]	48°C
		R	AAACTAGGATTAGATACCC		
	**16S**	AG	CGCCTGTTTATCAAAAACATGT	[18]	55°C
		BG	AGATCACGTAAGAATTTAATGGTC		
NUCLEAR	**Hexk**	HexF	AATGGGGAGCTTTCGGAGAT	[20]	48-50°C
		HexR	CATTGGCACAGTTTTGGTCAG		
	**EF1a**	M1F	GCWGCGGGTACTGGTGAR	modified from [21]	54°C
		M1R	ACACCWGTTTCAACWCGRCC		
		M51F	CTTCAGGATGTRTACAAAATTGGTG	modified from [22]	54°C
		M53R	GCAATATGAGCWGTGTGGCA		
	**H3**	HexAF	ATGGCTCGTACCAAGCAGACGGC	[23]	58°C
		HexAR	ATATCCTTGGGCATGATGGTGAC		
	**28S**	C1'	ACCCGCTGAATTTAAGCAT	[24]	55°C
		D2b	GTTAGACTCCTTGGTCCGT		
		C2'	GAAAAGAACTTTGRARAGAGAGT	[24]	54°C
		D3	ATAGTTCACCATCTTTCGGGTC	[25]	
	**18S**	A2	ATGGTTGCAAAGCTGAAAC	[26]	52°C
		9R	GATCCTTCCGCAGGTTCACCTA	[27]	
