# Supplementary material for: Secondary Sympatry Caused by Range Expansion Informs on the Dynamics of Microendemism in a Biodiversity Hotspot
Source: PLoS One. 2012 Nov 6;7(11):e48047. doi: 10.1371/journal.pone.0048047 (PMC3490955; doi:10.1371/journal.pone.0048047)
Supplement: Table S3 — Results of the Bayesian test of monophyly for Agnotecous species. (PDF) [file pone.0048047.s007.pdf]

TABLE S3

Monophyly constraint	Mitochondrial total	EF1a	28S	Nuclear total
A. meridionalis	27000 (100)	26843 (99.4)	27000 (100)	27000 (100)
A. clarus	27000 (100)	26264 (97.3)	26786 (99.2)	27000 (100)
A. obscurus	25882 (95.9)	18437 (68.3)	-	26879 (99.6)
A. albifrons	27000 (100)	26445 (97.9)	-	27000 (100)
A. tapinopus	27000 (100)	26988 (99.9)	27000 (100)	27000 (100)
A. azurensis	**0 ***	25399 (94.1)	27000 (100)	27000 (100)
A. yahoue	19670 (72.9)	26984 (99.9)	26821 (99.3)	27000 (100)
Agnotecous sp.	27000 (100)	26971 (99.9)	26999 (99.9)	27000 (100)
A. sarramea	18007 (66.7)	26747 (99.1)	26435 (97.9)	26975 (99.9)
Number total of trees	27000	27000	27000	27000
