# Supplementary material for: Secondary Sympatry Caused by Range Expansion Informs on the Dynamics of Microendemism in a Biodiversity Hotspot
Source: PLoS One. 2012 Nov 6;7(11):e48047. doi: 10.1371/journal.pone.0048047 (PMC3490955; doi:10.1371/journal.pone.0048047)
Supplement: Table S4 — Details of the geographic distribution of species used in the analysis of geographical pattern of speciation. (PDF) [file pone.0048047.s008.pdf]

TABLE S4

Species	Locality	Alt.	GPS details		Precision	Ref.
A. chopardi	Rivière Bleue	260 m	22°05'13,1"S	166°38'01,3"E	Haute Rivière Bleue	(1)
	Pic du Grand Kaori	260 m	22°16'47,1"S	166°53'39,9"E	-	(1)
A. doensis	Mont Do	950 m	21°45’02’’S	166°00’09’’E	-	(1)
A. sarramea	Col d'Amieu	680 m	21°36'14"S	165°46'27"E	Mé Aréto	(1)
		540 m	21°33'52"S	165°46'07"E	Table Unio	(1)
	Dogny	930 m	21°37'S	165°53'E	-	(2)
	Farino	400 m	21°37'S	165°46'E	-	(2)
A. tapinopus	Mont Mou	340 m	22°04'30"S	166°19'52"E	base	(1)
	Forêt de Thy	120 m	22°11'29"S	166°32'16,08"E	-	(1)
	Mont Koghis	-	*22°10'S*	*166°30'E*	Mt Bouo (W slope)	(3)
		510 m	22°10'44.00"S	166°30'31.00"E		(1)
	Yahoue	-	*22°12'S*	*166°29'E*	Yahoué village	(3)
A. humboldti	Rivière Ngoye	-	*21°52'S*	*166°25'E*	Near Mont Humboldt	(4)
A. nekando	Mont Nekando	-	*21°52'S*	*166°26'E*	-	(4)
A. occidentalis	Col des Rousettes	400 m	21°25'31,8"S	165°27'50,3"E	-	(1)
	Bourail	-	*21°33'S*	*165°29'E*	-	(1)
A. novaecaledoniae	Mt Rembai		*21°35'S*	*165°50'E*	-	(2)
A. albifrons	Gelima	730 m	21°36'S	165°58'E	-	(2)
	Farino	200 m	21°39'S	165°47'E	-	(2)
	Col d'Amieu	690 m	21°36'14"S	165°46'27"E	Table Unio	(1)
		550 m	21°33'52"S	165°46'07"E	Mé Aréto	(1)
		460 m	21°35'04,3"S	165°46'25,5"E	Col Toma	(1)
A. meridionalis	Port Boisé	20 m	22°20'54,2"S	166°58'05,9"E	-	(1)
		5 m	22°20'53,1"S	166°57'50,5"E	-	(1)
	Ile des Pins	20 m	22°35'21,7"S	167°31'19,4"E	-	(1)
		10 m	22°39'05,60"S	167°26'30,10"E	Kuto village	(5)
A. obscurus	Mont Panié	250 m	20°33'16"S	164°47'36"E	-	(1)
	Manjelia	700 m	20°24'0.70"S	164°31'41.00"E	-	(1)
	Touho	400 m	20°47'31.2"S	165°13'49.1"E	-	(2)
	Pic Amoa	500 m	20°58'S	165°17'E	-	(2)
	Houaïlou	-	*21°16'S*	*165°14'E*	40 km from W	(3)
	Poindimié	-	*20°53'S*	*165°13'E*	Vallée rivière Tiwaka	(3)
	Daewenia	620 m	164°40'51,23"E	20° 32' 15,89" S	-	(1)
	Wewec	380 m	164° 43' 50,28" E	20° 35' 55,44" S	-	(1)
	Wayen	590 m	164° 52' 17,26" E	20° 38' 23,69" S	-	(1)
	Aoupinié	790 m	21°10'52"S	165°18'06"E	-	(1)
A. clarus	Rivière Bleue	210 m	22°09'12"S	166°40'41,1"E	Rivière Blanche	(1)
	Pic du Grand Kaori	260 m	22°16'47,1"S	166°53'39,9"E	-	(1)
	Pic du Pin	290 m	22°14'52.50"S	166°49'42.60"E	-	(1)
A. yahoue	Mont Mou	390 m	22°04'30"S	166°19'52"E	-	(1)
	Monts Dzumac	880 m	22°1'45,7"S	166°28'13,2"E	-	(1)
	Mont Humboldt	1010 m	21°52’48.6"S	166°25’14"E	-	(1)
	Monts Koghis	510 m	22°10'44.00"S	166°30'31.00"E	-	(1)
		-	22°10'12,89"S	166°30'40,42"E	Mt Bouo (W slope)	(3)
	Yahoue	-	22°12'32,89"S	166°29'16,74"E	Yahoué village	(3)

A. azurensis	Rivière Bleue	175 m	22°07'11,3"S	166°39'34,1"E	Pont du mois de mai	(1)
		50 m	22°06’S	166°39’E	Kaori Géant	(2)
		180 m	22°7'40.86"S	166°39'49.26"E	-	(1)
		180 m	22°05'57,5"S	166°39'45,2"E	Gué de la Pourina	(1)
		210 m	22°09'12"S	166°40'41,1"E	Rivière Blanche	(1)
	Foret Nord	480 m	22°19'S	166°55'E	-	(2)
	Grand Kaori	260 m	22°16'47,1"S	166°53'39,9"E	-	(1)
	Port Boisé	-	*22°20'54,2"S*	*166°58'05,9"E*	-	(6)
	Pic du Pin	290 m	22°14'52.50"S	166°49'42.60"E	-	(1)
A. brachypterus	Sarraméa	180 m	*21°38'27,01"S*	*165°50'40,78"E*	Village	(6)
	Pocquereux	30 m	21° 44'03"S	165° 53'51"E	-	(1)
A. minoris	Mont Mou	390 m	22°04'30"S	165°19'52"E	Base	(4)
A. petchekara	Col de Petchekara	320 m	21°34’S	166°07’E	-	(4)
A. pinsula	Ile des Pins	20 m	22°35'21.7"S	167°31'19.4"E	-	(1)
A. robustus	Aoupinié	790 m	21°10'52"S	165°18'06"E	-	(1)
	Poindimié	-	*20°51'S*	*165°15'E*	Vallée Amoa	(6)
	Poya	-	*21°13'S*	*165°16'E*	South of Mont Aoupinié	(6)
	Mont Panié	-	*20°33'S*	*164°47'E*		(3)
Agnotecous sp.	Mont Mou	1140 m	22°3'42.4"S	166°20'50.3"E	Sommet	(1)
		1015 m	22°3'57.1" S	166°20'35.5"E	Maquis	(1)
