# Supplementary material for: Secondary Sympatry Caused by Range Expansion Informs on the Dynamics of Microendemism in a Biodiversity Hotspot
Source: PLoS One. 2012 Nov 6;7(11):e48047. doi: 10.1371/journal.pone.0048047 (PMC3490955; doi:10.1371/journal.pone.0048047)
Supplement: Table S5 — Results of the asymmetry tests conducted under SymmeTREE for seven test statistics. (PDF) [file pone.0048047.s009.pdf]

TABLE S5

statistic	M_R	I_C	M_{Π}*	M_{Π}	M_{Σ}*	M_{Σ}	B_1
Min ERM	1.30E-08	105	-1.4	1.30E-08	0.3	5.64	3.25
Max ERM	1	0	0	1	1	15	10.6667
Observed	0.49	53	-0.82	0.00021	0.56	10.34	6.83
P-value		0.016	0.0097	0.0066	0.011	0.0093	0.022
