# Supplementary material for: Secondary Sympatry Caused by Range Expansion Informs on the Dynamics of Microendemism in a Biodiversity Hotspot
Source: PLoS One. 2012 Nov 6;7(11):e48047. doi: 10.1371/journal.pone.0048047 (PMC3490955; doi:10.1371/journal.pone.0048047)
Supplement: Table S6 — Results of the diversification rate change test conducted under the R package apTreeshape. (PDF) [file pone.0048047.s010.pdf]

TABLE S6

Node	15	14	13	12	11	10	9	8	7	6	5	4	3	2
**P-value**	0.4991	0.4997	0.3801	0.6675	0.4261	0.6654	0.5003	0.2121	0.1076	0.4996	0.4862	0.4746	0.5289	0.293
**Delta 1**	0.3038	0.3038	0.5188	0.4742	-0.0687	0.4742	0.2882	-0.357	1.0562	0.124	0.1118	0.1016	0.0929	-1.0693
