# Supplementary material for: Secondary Sympatry Caused by Range Expansion Informs on the Dynamics of Microendemism in a Biodiversity Hotspot
Source: PLoS One. 2012 Nov 6;7(11):e48047. doi: 10.1371/journal.pone.0048047 (PMC3490955; doi:10.1371/journal.pone.0048047)
Supplement: Table S7 — Results of the BDL analyses and comparison of AIC values from different diversification models. (PDF) [file pone.0048047.s011.pdf]

TABLE S7

	Model	Number of parameters	Lh	AIC	AICc	Δ AICrc
Constant rate	Pure Birth	1	-5.44	12.87	13.16	3.19
	Birth Death	2	-5.44	14.87	15.79	5.82
Variable rate	y-2-rate	2	-4.52	15.05	15.97	6
