# Supplementary material for: Secondary Sympatry Caused by Range Expansion Informs on the Dynamics of Microendemism in a Biodiversity Hotspot
Source: PLoS One. 2012 Nov 6;7(11):e48047. doi: 10.1371/journal.pone.0048047 (PMC3490955; doi:10.1371/journal.pone.0048047)
Supplement: Text S1 — Additional information on sequencing protocols. (PDF) [file pone.0048047.s012.pdf]

Text S1 *Additional information on sequencing protocols*

DNA extractions from tissue samples were performed using a QIAamp DNA MicroKit (QIAGEN) following the manufacturer's instructions. Amplifications were made in a 25 µL volume reaction with 0.4 µL of each 10 pM/µl primers, 19.2 µL H_20, 2.5 µL buffer, 1.25 µL DMSO, 1 µL MIX dNTP, 0.15 µL Taq polymerase and 1 µL DNA extract. The oligonucleotide primers used for polymerase chain reaction (PCR) and sequencing are listed in Table S2. PCR reactions consisted of an initial denaturing step at 94 °C for 4 min, 40 amplification cycles (denaturation at 94 °C for 30 s, annealing between 48 and 55 °C for 40 s, and extension at 72 °C for 40 s) and a final step at 72 °C for 7 min. PCR products were checked on agarose gel and sequenced in both directions with the same primers at Table S2. Sequences were cleaned and coding sequences were translated using the invertebrate mitochondrial genetic code to check for the absence of stop codons using Sequencher (Gene Codes Corporation, v. 4.8 Build 3767, 2007). All genes were screened for potential contamination using the Blastx algorithm on GenBank (http://blast.ncbi.nlm.nih.gov/Blast.cgi). All newly generated sequences are deposited in GenBank (accession numbers and voucher references, see Table S1).
