# Supplementary material for: Secondary Sympatry Caused by Range Expansion Informs on the Dynamics of Microendemism in a Biodiversity Hotspot
Source: PLoS One. 2012 Nov 6;7(11):e48047. doi: 10.1371/journal.pone.0048047 (PMC3490955; doi:10.1371/journal.pone.0048047)
Supplement: Text S2 — Characteristics of mitochondrial (a) and nuclear (b) datasets used in this study. (PDF) [file pone.0048047.s013.pdf]

Text S2. *Characteristics of mitochondrial (a) and nuclear (b) datasets used in this study*

(a)

MITOCHONDRIAL				
Studied gene	Number of taxa	Sequence lenght (in base pairs)	Number of informative sites within the intra group (%)	Evolution model
16S	36	522	128 (24.5 %)	-
12S	32	408	43 (10.5 %)	-
Cytb	36	346	92 (26.6 %)	-
CO1	36	1272	224 (17.6 %)	-
CO2	26	400	54 (13.5 %)	-
Total	37	2948	541 (18.4 %)	GTR+G

(b)

NUCLEAR				
Studied gene	Number of taxa	Sequence lenght (in base pairs)	Number of informative sites within the intra group (%)	Evolution model
EF1-α	37	990	67 (6.8 %)	GTR+I+G
28S	28	1466	148 (10.1 %)	GTR+I+G
HexK	16	464	11 (2.4 %)	-
H3	33	329	10 (3 %)	-
18S	31	652	5 (0.8 %)	-
Total	37	3901	241 (6.2 %)	GTR+I+G
