# Supplementary material for: Secondary Sympatry Caused by Range Expansion Informs on the Dynamics of Microendemism in a Biodiversity Hotspot
Source: PLoS One. 2012 Nov 6;7(11):e48047. doi: 10.1371/journal.pone.0048047 (PMC3490955; doi:10.1371/journal.pone.0048047)
Supplement: Text S3 — Congruence tests between nuclear and mitochondrial data sets. (PDF) [file pone.0048047.s014.pdf]

Text S3. *Congruence tests between nuclear and mitochondrial data sets*

Discordance between gene trees and species trees can frequently occur through introgression or stochastic lineage sorting of ancestral hybridization. In particular, previous studies have shown that mitochondrial introgression is common in recent clades with rapid speciation [1], but this effect can be removed by the use of multiple and unlinked nuclear genes [2]. We thus sequenced five fragments of mitochondrial genes (CO1, CO2, Cytb, 12S, and 16S) and five fragments of nuclear genes (EF1α, 18S, 28S, H3, and Hexk). Mitochondrial introgression predicts that mitochondrial gene trees will contain as many or more non monophyletic species as do nuclear gene trees [2]. A Bayesian test of monophyly is a useful method for evaluating the monophyly of each species because posterior probabilities of nodes can be interpreted as the probability that those nodes exist [3]. This test is used to discard introgression occurrence in phylogeny [1,4,5]. For four data partitions (EF1α, 28S, mitochondrial total and nuclear total), we constructed in PAUP* 9 constrained trees corresponding to hypotheses of monophyly for each of the 9 species in which multiple populations have been sampled. For each data partition, monophyly constraints were used to filter the posterior distribution of trees using both runs from MrBayes, after removal of the burn-in. A hypothesis of monophyly is rejected if less than 5% of the trees from a given analysis were retained after filtering with a given constrained tree [1,5,6,7]. For each species, rejection of monophyly at the mtDNA locus with significant evidence for monophyly at nuclear loci will be interpreted as evidence of mitochondrial introgression [1].

According to the Bayesian tests of monophyly (Table S3), the null hypothesis of monophyly was rejected in mitochondrial data set for *A. azurensis* only, but confirmed for all the species in nuclear data set. Because these results suggest mitochondrial introgression, further analyses were restricted to nuclear data sets. Furthermore, the Bayesian and parsimony analyses

resulted in very similar relationships amongst taxa (electronic supplementary material, Fig. S1).
