# Supplementary material for: Secondary Sympatry Caused by Range Expansion Informs on the Dynamics of Microendemism in a Biodiversity Hotspot
Source: PLoS One. 2012 Nov 6;7(11):e48047. doi: 10.1371/journal.pone.0048047 (PMC3490955; doi:10.1371/journal.pone.0048047)
Supplement: Text S4 — Details about phylogenetic analyses. (PDF) [file pone.0048047.s015.pdf]

Text S4. *Details about phylogenetic analyses*

Four Markov chains were run simultaneously for 15 million generations sampling every 1000 generations to ensure independence of samples. The first 1.5 million generated trees (10%) were discarded as burn-in and determined empirically from the log-likelihood values using Tracer V1.4 [8]. The remaining trees were used to construct the 50% majority-rule consensus trees. Two independent runs were performed to check whether convergence on the same posterior distribution was reached and if final trees converged on the same topology. The statistical confidence in nodes was evaluated by posterior probabilities.

For parsimony analyses under TNT, the search strategy consisted first of 1000 replications of Random Addition Sequence and Tree Bisection and Reconnection (TBR). Then, to avoid local optima [9], we added 100 iterations of tree fusing [10], each iteration being swapped with TBR and Subtree Pruning and Regrafting (SPR); and 20 iterations of ratchetting [10], weighting characters with a factor of four. TNT has also been used to calculate Bootstrap support [BS; 11] values with 1000 replicates.
