# Supplementary material for: Secondary Sympatry Caused by Range Expansion Informs on the Dynamics of Microendemism in a Biodiversity Hotspot
Source: PLoS One. 2012 Nov 6;7(11):e48047. doi: 10.1371/journal.pone.0048047 (PMC3490955; doi:10.1371/journal.pone.0048047)
Supplement: Text S5 — Additional BEAST parameters and details of calibrations. (PDF) [file pone.0048047.s016.pdf]

Text S5. *Additional BEAST parameters and details of calibrations*

We assigned the best fitting model, as estimated by jMODELTEST v 0.1.1 [12] , using the whole data set. Substitution rates were estimated using an uncorrelated lognormal relaxed molecular clock model and the Yule process of speciation was assumed, as recommended for species-level phylogenies [13].

We used a normal distribution for the tree prior to calibrate node with a standard deviation of 1%, which is particularly suitable in secondary calibration points, as it reflects uncertainty date estimates [14]. All relationships within *Agnotecous* were left free to vary so that topological uncertainty was incorporated into posterior estimates of divergence dates. We confirmed the analysis with two independent runs of 25 millions of generations, and sampled each 2500 generations to obtain a maximum of 10000 samples. Both analyses converged on very similar posterior estimates. We used TRACER version 1.4.1 [8] to determine convergence, measure the effective sample size of each parameter, and calculate mean and 95% highest posterior density interval (HPD) for divergence times. We checked if a reasonable effective sample size greater than 200 was achieved for all parameters after the analyses, as recommended by [13]. Results of the two runs were combined with LogCombiner v1.4.7 [13] and the consensus tree compiled with TreeAnnotator v1.4.7 [13].
