# Supplementary material for: Secondary Sympatry Caused by Range Expansion Informs on the Dynamics of Microendemism in a Biodiversity Hotspot
Source: PLoS One. 2012 Nov 6;7(11):e48047. doi: 10.1371/journal.pone.0048047 (PMC3490955; doi:10.1371/journal.pone.0048047)
Supplement: Text S6 — Details of the rate.estimate function used in GEIGER. (PDF) [file pone.0048047.s017.pdf]

Text S6. *Details of the rate.estimate function used in GEIGER*

```
library(ape)
library(geiger)
tr<-read.nexus("Agnotecous-dating.trees")
plot(ladderize(tr),type="p", use.edge.length=FALSE, no.margin=TRUE, cex = 0.8,
label.offset=0.4)
tip <- c("Adom", "LsaV", "Egu", "NviS")
plot(drop.tip(tr, tip))
tr<-drop.tip(tr, tip)
tip <- c("Adom", "LsaV", "Egu", "NviS", "A-albifrons", "A-obscurus", "A-meridionalis", "A-
clarus", "A-robustus")
tr19<-drop.tip(tr, tip)
tip <- c("Adom", "LsaV", "Egu", "NviS", "A-albifrons", "A-obscurus", "A-meridionalis", "A-
clarus", "A-robustus", "A-tapinopus")
tr20<-drop.tip(tr, tip)
tip <- c("Adom", "LsaV", "Egu", "NviS", "A-albifrons", "A-obscurus", "A-meridionalis", "A-
clarus", "A-robustus", "A-tapinopus", "A-chopardi")
tr21<-drop.tip(tr, tip)
tip <- c("Adom", "LsaV", "Egu", "NviS", "A-albifrons", "A-obscurus", "A-meridionalis", "A-
clarus", "A-robustus", "A-tapinopus", "A-chopardi", "A-azurensis")
tr22<-drop.tip(tr, tip)
tip <- c("Adom", "LsaV", "Egu", "NviS", "A-albifrons", "A-obscurus", "A-meridionalis", "A-
clarus", "A-robustus", "A-tapinopus", "A-chopardi", "A-azurensis", "A-yahoue")
tr23<-drop.tip(tr, tip)
tip <- c("Adom", "LsaV", "Egu", "NviS", "A-albifrons", "A-obscurus", "A-meridionalis", "A-
clarus", "A-robustus", "A-tapinopus", "A-chopardi", "A-azurensis", "A-yahoue", "A-
brachypterus", "A-minoris", "A-yahoue-like")
tr24<-drop.tip(tr, tip)
tip <- c("Adom", "LsaV", "Egu", "NviS", "A-albifrons", "A-obscurus", "A-meridionalis", "A-
clarus", "A-robustus", "A-tapinopus", "A-chopardi", "A-azurensis", "A-yahoue", "A-
brachypterus", "A-minoris", "A-yahoue-like", "A-pinsula")
tr25<-drop.tip(tr, tip)
tip <- c("Adom", "LsaV", "Egu", "NviS", "A-albifrons", "A-obscurus", "A-meridionalis", "A-
clarus", "A-robustus", "A-tapinopus", "A-chopardi", "A-azurensis", "A-yahoue", "A-
brachypterus", "A-minoris", "A-yahoue-like", "A-pinsula", "A-sarramea")
tr26<-drop.tip(tr, tip)
tip <- c("Adom", "LsaV", "Egu", "NviS", "A-albifrons", "A-obscurus", "A-meridionalis", "A-
clarus", "A-robustus", "A-tapinopus", "A-chopardi", "A-azurensis", "A-yahoue", "A-pinsula",
"A-sarramea", "A-doensis", "A-occidentalis")
tr27<-drop.tip(tr, tip)
tip <- c("Adom", "LsaV", "Egu", "NviS", "A-albifrons", "A-obscurus", "A-meridionalis", "A-
clarus", "A-robustus", "A-tapinopus", "A-chopardi", "A-azurensis", "A-yahoue", "A-pinsula",
"A-sarramea", "A-doensis", "A-occidentalis", "A-brachypterus")
tr28<-drop.tip(tr, tip)
tip <- c("Adom", "LsaV", "Egu", "NviS", "A-robustus", "A-tapinopus", "A-chopardi", "A-
azurensis", "A-yahoue", "A-brachypterus", "A-yahoue-like", "A-minoris", "A-pinsula", "A-
sarramea", "A-sarramea", "A-occidentalis", "A-doensis", "A-clarus", "A-meridionalis")
tr30<-drop.tip(tr, tip)
```

```
tip <- c("Adom", "LsaV", "Egu", "NviS", "A-robustus", "A-tapinopus", "A-chopardi", "A-azurensis", "A-yahoue", "A-brachypterus", "A-yahoue-like", "A-minoris", "A-pinsula", "A-sarramea", "A-sarramea", "A-occidentalis", "A-doensis", "A-obscurus", "A-albifrons")
tr30<-drop.tip(tr, tip)
rate.estimate(phy=tr28, epsilon = 0, missing = 3)
rate.estimate(phy=tr, epsilon = 0.5, missing = 3)
rate.estimate(phy=tra, epsilon = 0, missing = 3)
rate.estimate(phy=trb, epsilon = 0, missing = 3)
rate.estimate(phy=trb2, epsilon = 0, missing = 3)
rate.estimate(phy=tr, epsilon = 0.5, missing = 3)
rate.estimate(phy=tra, epsilon = 0.5, missing = 3)
rate.estimate(phy=trb, epsilon = 0.5, missing = 3)
rate.estimate(phy=trb2, epsilon = 0.5, missing = 3)
```
